# Translations

**Published:** 1876-06

**Authors:** 


### Translations.

### MEDICINE AND THE FINE ARTS.

(*From the German of Rohlf's Geschichte der Deutschen Medicin.*)

By JAMES I. TUCKER, A.M., M.D., Chicago.

All great poets have taken a lively interest in medicine. We know little of Shakspeare in this connection, but it is safe to say that he studied medicine as well as the old poets, for it is evinced by the masterly delineations in Lear, Hamlet and Macbeth. Concerning Goethe it is well known that he preferred the society of physicians, and neglected his legal studies, for a long time to such an extent that he attended only the medical college. In his autobiography he says of himself: "I gave to the study of jurisprudence only so much time and attention as was necessary to graduate with honor. Medicine fascinated me, for it enabled me to see Nature where she was not openly revealed." That Goethe's medical studies were not fruitless is shown by his discovery, later, of the intermaxillary bones and the metamorphosis of plants, which up to that time had been overlooked by anatomists and botanists. Lessing matriculated in medicine at Wittenberg, and later, was a diligent student of obstetrics at Leipsig, devoting his attention upon entering, to the history of syphilis. It is then, a fact, that great poets feel drawn towards medicine, and great physicians towards poetry, and the fine arts. Raphael

Frankenstein in his book entitled "The Poet and the Physician," has done full justice to this subject. How little great physicians have limited themselves to the study of nature and disease may be learned from the answer of Sydenham. Being asked what book the physician should read, he replied, "Don Quixote." It has been said that Raphael in his celebrated painting of the Faculty, utterly ignored the physicians. Marx was the first to remove this reproach. "But what," said he, (*Beitrage S.* 178) "if it should appear that only a superficial glance at the picture, and not the picture itself, should bear the burden of the ill humor and the sickly subserviency to rank? That another explanation should reveal that the physician, instead of being neglected and insulted by it, receives only praise and honor?"

Let us look at the painting of the poets and muses, with Apollo playing upon a sweet instrument, all together in the laurel grove, and wearing the expression of the profoundest ease and contentment, those who are listening to the music, as well as those who are entertaining themselves with conversation, exhibiting no trace of excitement or passion, then we can only believe that they are thinking of purity, goodness, truth, and value only the quieter virtues.

What could more appropriately represent the physician who with his whole soul devotes his knowledge and power to human woe, rendering to every one who needs it, self-abnegation, relief and all the blessings of the healing art.

Consequently in Art, as Raphael presented his higher conceptions for the benefit of mankind, so the medical profession becomes an imperishable glorification. To be permitted to behold in the picture of Parnassus the sentiment of the art of medicine must, therefore, inspire in every disciple the feeling of joy and pride.

### MEDICINE AND POETRY.

(*Translated from Rohlf's Geschichte der Deutschen Medicin. Stuttgart, 1875.*)

BY JAMES I. TUCKER, M.A., M.D., CHICAGO.

Medicine and poetry are the oldest of the arts. Poetry is the language of the childhood of a people, because the language of feeling, and its development precedes that of the understanding. The language of understanding is prose. We may study the history of every nation, search after the most ancient traditions of Greece and the Indus, of the Scandinavians and the Indians, everywhere we find that the earliest products of the mind are expressed in metrical form. Hence a people can no longer produce a great poet after it has passed the period of popular sentiment and primitiveness; thereafter the understanding rules supreme and the *blastemal* stage is begun. A truly great poet must possess a childlike feeling, for in such only can fancy take root. In relation to age is it, therefore, possible to divide the arts into those which unfold themselves during the childhood and those which develop in the maturity of a nation's existence. To the first belong poetry and medicine, to the latter sculpture and painting. Between the two, stands music. To communicate his feelings and thoughts to others; to sing the deeds of heroes, gods and demigods; to heal the wounded—these, if indeed the germs exist and materials for the realization of the idea, these naturally precede those which are to come. With poetry it was different; here, in the very beginning, a medium of communication was given, namely, language. Thus also with medicine. Nature indicated the manner in which wounds must be healed, and how in fevers attending internal disease, recovery must take place through her inherent power. And as Apollo was regarded by the Greeks as the god alike of the poet and the physician, so those who distinguished themselves by the healing of sicknesses, were held by their fellow-men to be beings endowed by superior gifts, and like Chiron the centaur and Esculapius, to be the sons of Apollo. It is not by accident that Greek medicine attained to its high degree of perfection. The principal reason is, that its physicians comprehended *the principles of their art.* In all Grecian medicine—and Hippocrates, even, spoke of a medicine still more ancient—we find no where and at no time an effort to convert the art into a science, as it is *the persistent tendency of modern medicine to do.* To the Greeks, medicine was the *healing art.* We observe, therefore, that all physicians deservedly prominent who have followed Hippocrates, from antiquity to the present day, have held the arts in high esteem, having been interested in music, sculpture or painting, perhaps participated in them, and showed, for poetry, the art of arts, and consequently for all the humane belletristical studies, the most devoted consideration.

